# Model based on five tumour immune microenvironment-related genes for predicting hepatocellular carcinoma immunotherapy outcomes

**DOI:** 10.1186/s12967-020-02691-4

**Published:** 2021-01-06

**Authors:** Xinyu Gu, Jun Guan, Jia Xu, Qiuxian Zheng, Chao Chen, Qin Yang, Chunhong Huang, Gang Wang, Haibo Zhou, Zhi Chen, Haihong Zhu

**Affiliations:** State Key Laboratory for Diagnosis and Treatment of Infectious Diseases, National Clinical Research Center for Infectious Diseases, Collaborative Innovation Center for Diagnosis and Treatment of Infectious Diseases, The First Affiliated Hospital, College of Medicine, Zhejiang University, NO. 79 Qingchun Road, Hangzhou, 310003 Zhejiang China

**Keywords:** HCC, Risk model, Immune environment, Prognosis, Immunotherapy

## Abstract

**Background:**

Although the tumour immune microenvironment is known to significantly influence immunotherapy outcomes, its association with changes in gene expression patterns in hepatocellular carcinoma (HCC) during immunotherapy and its effect on prognosis have not been clarified.

**Methods:**

A total of 365 HCC samples from The Cancer Genome Atlas liver hepatocellular carcinoma (TCGA-LIHC) dataset were stratified into training datasets and verification datasets. In the training datasets, immune-related genes were analysed through univariate Cox regression analyses and least absolute shrinkage and selection operator (LASSO)-Cox analyses to build a prognostic model. The TCGA-LIHC, GSE14520, and Imvigor210 cohorts were subjected to time-dependent receiver operating characteristic (ROC) and Kaplan–Meier survival curve analyses to verify the reliability of the developed model. Finally, single-sample gene set enrichment analysis (ssGSEA) was used to study the underlying molecular mechanisms.

**Results:**

Five immune-related genes (*LDHA*, *PPAT*, *BFSP1*, *NR0B1,* and *PFKFB4*) were identified and used to establish the prognostic model for patient response to HCC treatment. ROC curve analysis of the TCGA (training and validation sets) and GSE14520 cohorts confirmed the predictive ability of the five-gene-based model (AUC > 0.6). In addition, ROC and Kaplan–Meier analyses indicated that the model could stratify patients into a low-risk and a high-risk group, wherein the high-risk group exhibited worse prognosis and was less sensitive to immunotherapy than the low-risk group. Functional enrichment analysis predicted potential associations of the five genes with several metabolic processes and oncological signatures.

**Conclusions:**

We established a novel five-gene-based prognostic model based on the tumour immune microenvironment that can predict immunotherapy efficacy in HCC patients.

## Background

Hepatocellular carcinoma (HCC) is a highly malignant cancer and ranks as the third leading cause of cancer-related deaths worldwide [1]. The 5‐year survival and overall survival (OS) rates are below 12%. Precursors of most HCC cases include liver cirrhosis, chronic hepatitis viral infections, alcohol-related liver disease, non-alcoholic fatty liver disease, and drug-induced hepatitis. As HCC is usually diagnosed at advanced stages [2], it can be difficult to treat. Thus, it is important to elucidate the molecular mechanisms underlying HCC progression and develop novel therapeutic targets to improve patient survival outcomes.

The immune microenvironment plays a critical role in tumorigenesis and is correlated with tumour progression and treatment efficacy [3, 4]. Systemic immune therapeutics have shown efficacy against HCC, especially for patients without an opportunity to undergo resection or liver transplantation [2, 5]. Common immunotherapy strategies include chimeric antigen receptor-engineered T cells (CAR-T cells), cancer vaccines, cytokine therapy, and immune checkpoint inhibitors (ICIs). Currently, ICIs are the most successful class of immune therapeutics, both as monotherapy and combination therapy [6]. For example, the efficacy of anti-programmed cell death 1 (anti-PD-1) and anti-programmed cell death ligand 1 (anti-PD-L1) in HCC has been investigated. PD-L1 expressed by T cells regulates immune responses at the initiation phase in lymph nodes and at the effector phase in tumour cells [7]. The restoration of function in “exhausted” T cells and the depletion of immunosuppressive regulatory T lymphocytes using monoclonal antibodies targeting these receptors have opened up new avenues for the treatment of several malignancies [8]. However, only approximately 25% of HCC patients with high infiltration of PD-1-expressing T cells respond to ICIs [9], and identification of patients who will respond well to ICIs is challenging.

Conventional strategies using tumour-node-metastasis (TNM) classification to predict HCC prognosis can help guide decisions in HCC clinical therapy [10]. However, their predictive efficacies are less than satisfactory. Notably, the use of genome-sequencing technologies coupled with bioinformatics analyses has improved tumour diagnosis and prognosis capabilities. Gene-based prognostic models have been established to identify differential mRNA expression patterns between cancer and normal tissues. Datasets reporting the expression profiles of long noncoding RNAs (lncRNAs) [11], genes that regulate epigenetic modifications [12], and immune-related genes [13] from The Cancer Genome Atlas (TCGA) and NCBI Gene Expression Omnibus (GEO) databases have been increasingly explored to study disease prognosis. However, there is no mature model that can stably predict patient response to HCC immunotherapy and treatment outcome. Therefore, we established a novel five-gene-based model pertaining to the immune microenvironment and conducted bioinformatics analyses to assess the ability of this model to predict HCC immunotherapy outcomes.

In the present study, we conducted univariate Cox regression analyses and least absolute shrinkage and selection operator (LASSO)-Cox regression analyses to build a risk model. Risk score, time-dependent receiver operating characteristic (ROC) AUC, and Kaplan–Meier survival analyses were used to assess the prognostic ability of the model. The results indicate that our model can effectively predict the efficacy of immunotherapy and that the five genes can serve as potential independent biomarkers in clinical applications. Functional enrichment analysis predicted the potential associations of the upregulated and downregulated genes identified in our model with relevant biological mechanisms. Conclusively, we established a five-gene-based model based on the influence of the tumour immune microenvironment that could potentially be applied to predict patient response to HCC immunotherapy.

## Methods

### Data collection

Data from a total of 365 HCC patient samples from the TCGA-liver hepatocellular carcinoma (LIHC) dataset were retrieved and used for the analysis of prognostic gene expression signatures and the development of a prognostic model. Genes were excluded if corresponding patient sample data were lacking. Random sampling with arrangement was performed, wherein the 365 samples (including the training set [n = 219] and validation set [n = 146]) were randomly sampled 100 times with replacement. There was no significant difference in TNM stage, grade, OS, sex, or age between the training set and validation set (*p* > 0.05). The clinical data and mRNA expression data of the GSE14520 dataset (n = 221) were retrieved from the NCBI GEO database (https://www.ncbi.nlm.nih.gov/geo/), and an immunotherapy dataset (Imvigor210) was obtained from published study [14]. All patient data that were used in the present study had complete clinical information, including TNM stage, grade, survival time, sex, age, and immune-related gene expression.

### Establishment of the five-gene model based on immune-related genes

First, we screened for immune-related genes associated with HCC prognosis. The immune-related genes involved in HCC pathogenesis were collected from previous studies [15–17]. In the TCGA-LIHC training set, immune-related gene and survival data were analysed by univariate Cox regression analysis using the R package “coxph”. The criterion of *p* < 0.001 was selected as the filtering threshold. We screened the immune-related genes associated with HCC prognosis. Second, a LASSO-Cox regression analysis was used to further filter the prognostic genes[18]. This method enables the simultaneous selection of variables and parameter estimation and can better solve the multicollinearity problem in regression analyses [18]. A prognostic gene signature was established based on the LASSO-Cox regression model coefficients (β values) multiplied by the mRNA expression level. The risk score = [0.307 × mRNA expression level of *LDHA*] + [0.268 × mRNA expression level of *PPAT*] + [0.455 × mRNA expression level of *BFSP1*] + [0.234 × mRNA expression level of *NR0B1*] + [0.109 × mRNA expression level of *PFKFB4*].

### Assessment of the five-gene-based model by tissue microarray (TMA) analysis

To assess the prognostic ability of the five-gene-based model, we constructed a TMA comprised of 90 carcinoma tissues from HCC patients (Shanghai Outdo Biotech Co. Ltd., Shanghai, China) according to a reference method that was described previously [19]. Subsequently, immunohistochemistry (IHC) and integrated optical density (IOD) analyses were performed as described previously [20]. The primary antibodies used are shown in Additional file 1: Table S1. The IHC scores were obtained from independent assessments by three senior pathologists without any prior knowledge of patient characteristics. The IHC score of each patient was calculated using the following formula: IHC score = [0.307 × protein expression level of LDHA] + [0.268 × protein expression level of PPAT] + [0.455 × protein expression level of BFSP1] + [0.234 × protein expression level of NR0B1] + [0.109 × protein expression level of PFKFB4]. A Kaplan–Meier log-rank analysis was used to evaluate the difference in survival rates between the groups with a high IHC score and low IHC score.

### Validation of the performance and prognostic ability of the five-gene-based model

Time-dependent ROC analyses and Kaplan–Meier log-rank tests were used to evaluate the performance and prognostic ability of the model using verification datasets from the TCGA-LIHC dataset, the entire TCGA-LIHC dataset, the GSE14520 cohort, and the immunotherapy dataset (Imvigor210).

### Functional enrichment analyses

To explore the underlying molecular mechanisms of the five-gene-based model, single-sample gene set enrichment analysis (ssGSEA) of the gene expression profiles was used to identify the Kyoto Encyclopedia of Genes and Genomes (KEGG) pathways predicted to be correlated with the risk score [21, 22]. Clustering analyses were performed by gene set variation analysis (GSVA). A *p* < 0.05 and false discovery rate (FDR) < 0.05 were considered statistically significant.

### Statistical analysis

Statistical analyses were performed using SPSS v25 (IBM, Chicago, IL, USA), GraphPad Prism 7.0 (GraphPad Software, La Jolla, CA, USA), and R software (version 3.5.1). Student's *t*-test was used for statistical comparisons, the Kaplan–Meier method was used to estimate OS, and *p* < 0.05 was considered statistically significant.

## Results

### Identification of HCC prognostic gene expression signatures to construct the HCC prognostic model

**Fig. 1:**
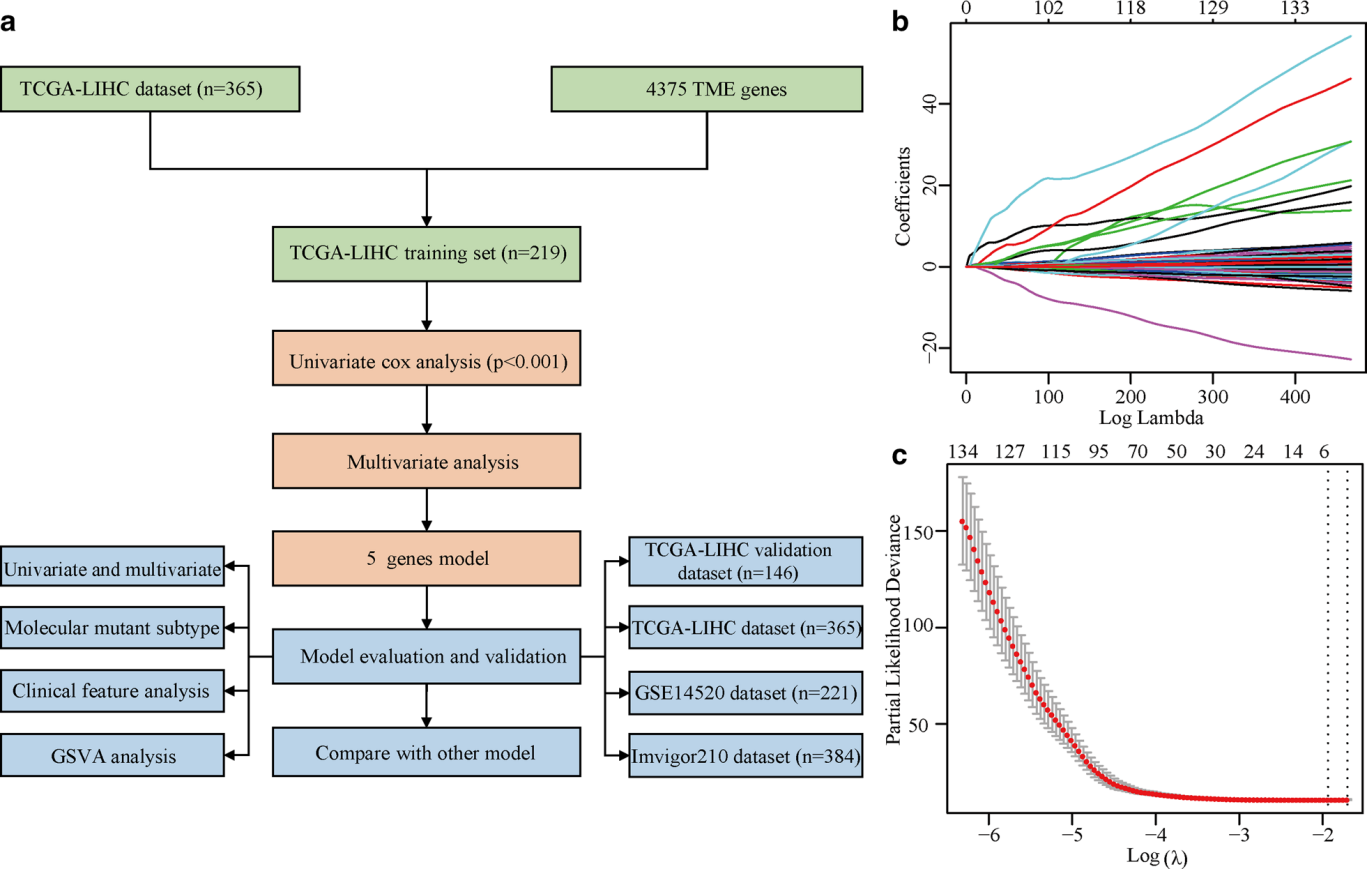
Overall analysis workflow and LASSO model profile plots for the potential prognostic indicators of HCC. a. Schematic flowchart of the workflow performed to build and validate the HCC prognostic model. b. LASSO coefficient profile plots of the 245 prognostic genes showing that the variations in the size of the coefficients of parameters shrink with an increasing value of the k penalty. c. Penalty plot for the LASSO model for the 245 prognostic genes with error bars denoting the standard errors. *LASSO* least absolute shrinkage and selection operator, *HCC* hepatocellular carcinoma, *OS* overall survival

A flowchart of the analysis workflow is illustrated in Fig. 1a. Using the TCGA-LIHC training set, univariate Cox regression analysis of the screening results, including 4,227 immune-related genes, led to the identification of 245 immune-related genesas potential prognostic indicators of HCC OS.

### Construction of the five-gene-based HCC prognostic model

After primary filtering, a LASSO-penalized Cox analysis was performed to further narrow down the mRNA expression profiles (Fig. 1b and c). Five genes were identified: lactate dehydrogenase A (*LDHA*), phosphoribosyl pyrophosphate amidotransferase (*PPAT*), beaded filament structural protein 1 (*BFSP1*), nuclear receptor subfamily 0 group B member 1 (*NR0B1*), and 6-phosphofructo-2-kinase/fructose-2,6-bisphosphatase 4 (*PFKFB4*). A stepwise multivariate Cox regression analysis was then performed to establish the prognostic model. The risk score was calculated by summing the weighted gene expression level of each of the five genes multiplied by their respective LASSO coefficients: risk score = [0.307 × mRNA expression level of *LDHA*] + [0.268 × mRNA expression level of *PPAT*] + [0.455 × mRNA expression level of *BFSP1*] + [0.234 × mRNA expression level of *NR0B1*] + [0.109 × mRNA expression level of *PFKFB4*]. All five genes showed positive LASSO coefficients in the multivariate Cox regression analysis. Next, the five-gene-based model was further evaluated for stability and reliability.

### Evaluation of the predictive efficacy of the five-gene-based model using the TCGA-LIHC training set

**Fig. 2:**
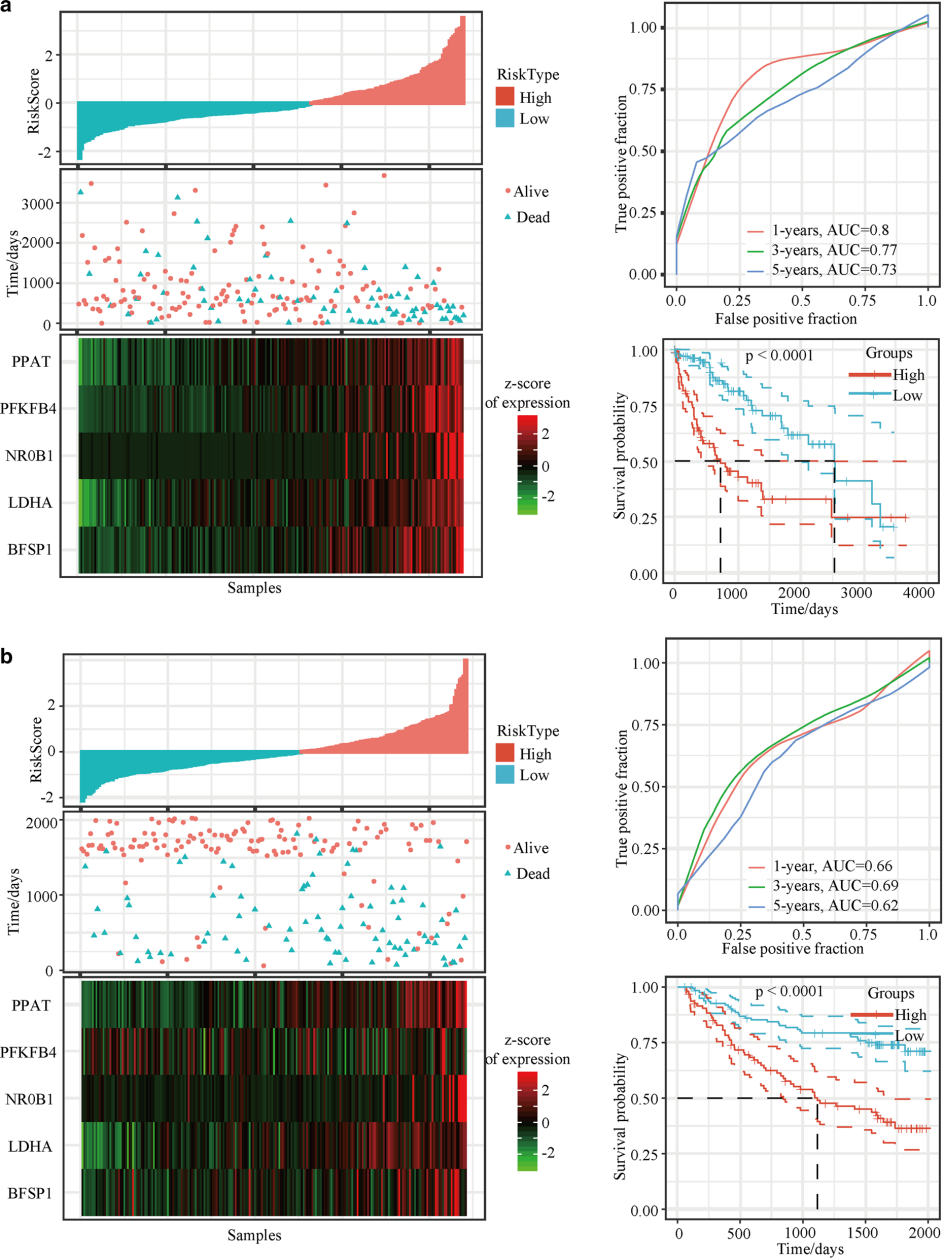
Output profiles from the analyses of the TCGA and GSE dataset based on the prognostic model. Risk score (top left), patient status (top right), mRNA expression heatmap (bottom left), and time-dependent ROC and Kaplan–Meier curves (bottom right) of the five-gene-based model for the a. TCGA-LIHC training set and b. GSE14520 dataset. *TCGA* The Cancer Genome Atlas, *ROC* receiver operating characteristic, *PPAT* phosphoribosyl pyrophosphate amidotransferase, *BFSP1* beaded filament structural protein 1, *LDHA* lactate dehydrogenase A, *NR0B1* nuclear receptor subfamily 0 group B member 1, *PFKFB4* 6-phosphofructo- 2-kinase/fructose-2,6-bisphosphatase 4

To determine the association between the gene expression signatures of these five genes and HCC patient survival outcome, risk scores (AUC values) were calculated with the five-gene-based model for each sample separately, and the optimal cut-off for the risk score was defined using the TCGA-LIHC training set (Fig. 2a). Higher AUC values indicated better classification performance of the five-gene-based HCC prognostic model. AUC values of 0.80, 0.77 and 0.73 were obtained for the 1-year, 3-year and 5-year survival rates, respectively. Kaplan–Meier survival analysis revealed that patients in the high-risk group showed worse prognosis than patients in the low-risk group (*p* < 0.0001; Fig. 2a). Taken together, these results indicate good performance of the established five-gene-based model for predicting HCC survival outcomes.

### Validation of the five-gene-based HCC prognostic model using the GSE14520 dataset, TCGA-LIHC testing set, and whole TCGA-LIHC dataset

To validate the stability of the five-gene-based model, a similar workflow was employed for the training set, wherein three datasets (the GSE14520 dataset, TCGA-LIHC testing set, and whole TCGA-LIHC dataset) were analysed. Risk scores for the five-gene-based model were obtained by calculating the AUC values of the respective ROC curves. AUC values of 0.75, 0.73 and 0.67 were obtained for 1-year, 3-year and 5-year survival rates, respectively, for the GSE14520 validation dataset. The results indicated that patients in the high-risk group showed significantly worse survival rates than patients in the low-risk group (*p* < 0.001; Fig. 2b). Consistent with the results of the GSE14520 dataset, patients in the high-risk group showed poorer OS than patients in the low-risk group (all *p* < 0.05) and the AUC values were above 0.6 for the whole TCGA-LIHC validation dataset and the TCGA-LIHC dataset (Additional file 2: Figure S1a and b). The results collectively showed that the five-gene-based model could predict patient survival duration based on gene expression levels.

### Upregulated genes (LDHA, PPAT, BFSP1, NR0B1, and PFKFB4) identified from the IHC TMA analysis predict poor prognosis

**Fig. 3:**
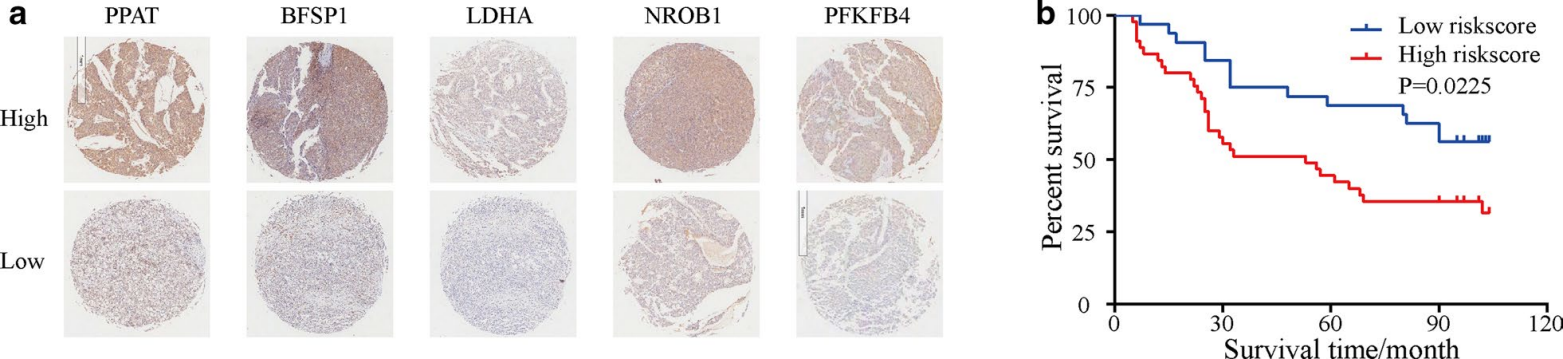
The expression levels of the 5 genes in tumour tissues linked to prognosis of HCC. a. IHC images of the expression of proteins included in the five-gene-based model in HCC tumour tissues in the tissue microarray. b. Kaplan–Meier analysis of the five-gene-based model for patients with low- or high-risk scores. *PPAT* phosphoribosyl pyrophosphate amidotransferase, *BFSP1* beaded filament structural protein 1, *LDHA* lactate dehydrogenase A, *NR0B1* nuclear receptor subfamily 0 group B member 1, *PFKFB4* 6-phosphofructo-2-kinase/fructose-2,6-bisphosphatase 4, *IHC* immunohistochemistry, *HCC* hepatocellular carcinoma

Next, the protein expression levels of the five genes *LDHA*, *PPAT*, *BFSP1*, *NR0B1* and *PFKFB4* were determined with an HCC TMA using IHC. Notably, we found that the protein levels of the five genes were significantly different in HCC tissues (Fig. 3a). The IHC score was calculated using the following formula: IHC score = [0.307 × protein expression level of LDHA] + [0.268 × protein expression level of PPAT] + [0.455 × protein expression level of BFSP1] + [0.234 × protein expression level of NROB1] + [0.109 × protein expression level of PFKFB4]. The group with high IHC scores was associated with higher protein expression levels and poorer disease prognosis than the group with low IHC scores (Fig. 3b). Variables that showed statistical significance in the univariate analysis were included in the Cox multivariate survival regression analysis. The results showed that the five-gene-based model score, grade and TNM stage were statistically significant (*p* < 0.05; Table 1). Thus, the results suggest that high protein expression of the five genes predicts poor disease prognosis.

**Table 1:** Univariate and multivariate analyses of factors correlated with overall survival

Variables	Univariate analysis			Multivariate analysis		
	HR	95%CI	p value	HR	95%CI	p value
Five-gene-based model	2.054	1.087–3.882	0.027	2.19	1.152–4.163	0.017
Sex	1.499	0.463–4.847	0.499			
Grade	1.936	1.062–3.529	0.031	1.878	1.024–3.443	0.042
Age	1.442	0.779–2.668	0.244			
TNM stage	1.792	1.054–3.046	0.031	1.79	1.033–3.103	0.038

*HR* hazard ratio, *CI* confidence interval, *TNM* tumour-node-metastasis

### Assessment of the ability of the five-gene-based model to predict immunotherapy efficacy

**Fig. 4:**
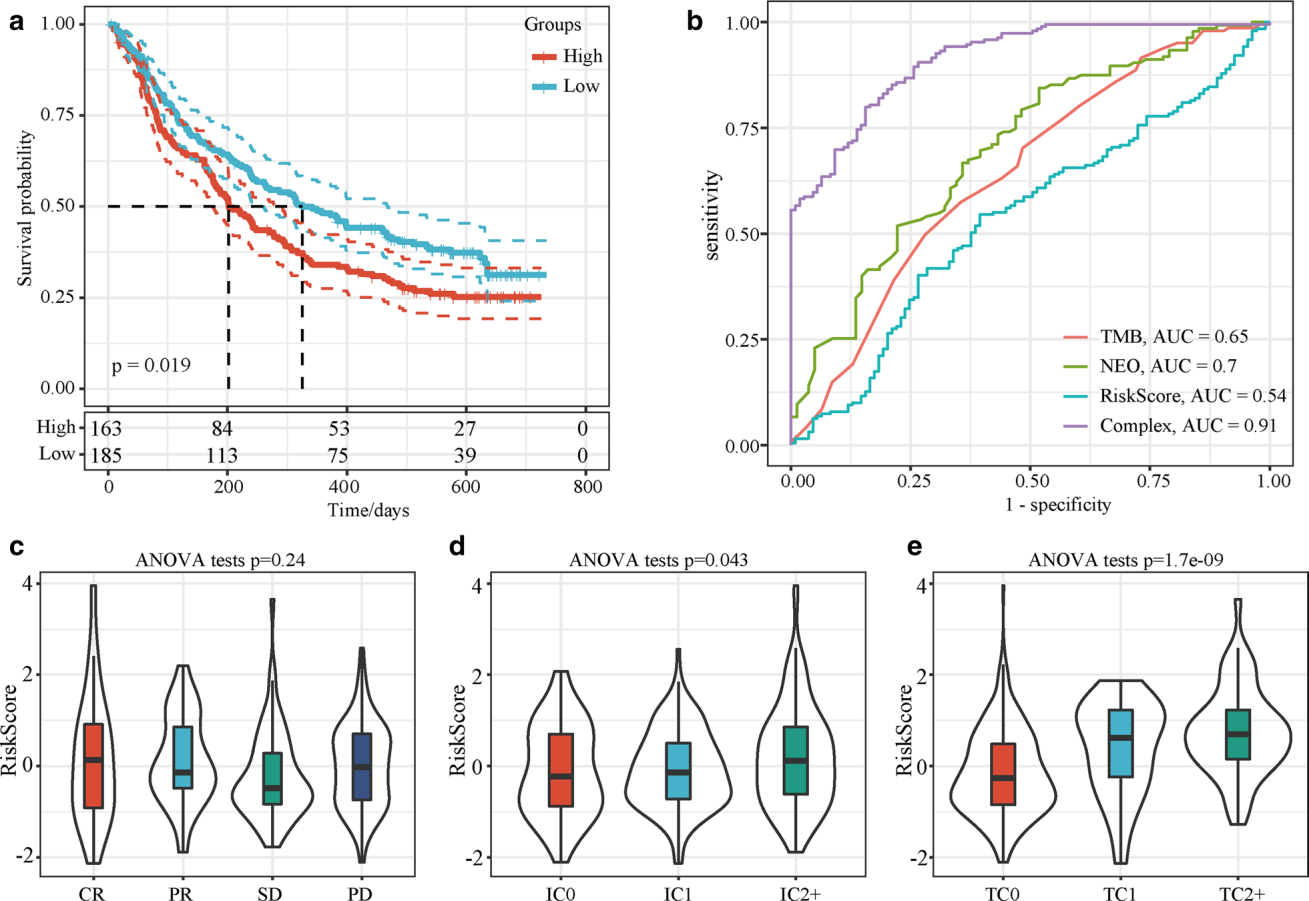
The five-gene-based model can predict immunotherapy efficacy for the Imvigor210 dataset. **a** Kaplan–Meier analysis of the five-gene-based model. **b** ROC curves of TMB, NEO burden, risk score, and the combination (NEO burden, TMB and risk score). The risk score was grouped by **c** immunotherapy efficacy, **d** immune cell subgroups, and e. tumour cell subgroups. *ROC* receiver operating characteristic, *NEO* neoantigens, *TMB* tumour mutational burden, *CR* complete response, *PR* partial response, *SD* stable disease, *PD* progressive disease, *IC* immune cell, *TC* tumour cell

Biomarkers that can effectively predict the efficacy of immunotherapy drugs are currently lacking. Thus, identifying new predictive markers is necessary to further improve precision immunotherapy. A transcriptome dataset (Imvigor210) of the treatment response data of patients who underwent anti-PD-L1 immunotherapy was retrieved to assess the ability of the five-gene-based model to predict immunotherapy efficacy. Kaplan–Meier analysis showed that a high-risk score was associated with a poorer survival rate than a low risk score (Fig. 4a). ROC curve analysis showed that the combined consideration of neoantigen (NEO) burden, tumour mutational burden (TMB) and risk score output a higher AUC value (AUC = 0.91) than NEO burden (AUC = 0.7), TMB (AUC = 0.65), or risk score (AUC = 0.54) alone (Fig. 4b). The risk score was not correlated with the immunotherapy efficacy subgroup (*p* > 0.05; Fig. 4c) but was correlated with the immune cell and tumour cell subgroups (*p* < 0.05; Fig. 4d and e). These results suggest that considering the five-gene-based model score together with NEO burden and TMB could enhance the assessment of immunotherapy efficacy and identify patients who will respond to immunotherapy.

### The five-gene-based model can predict different clinical characteristics

**Fig. 5:**
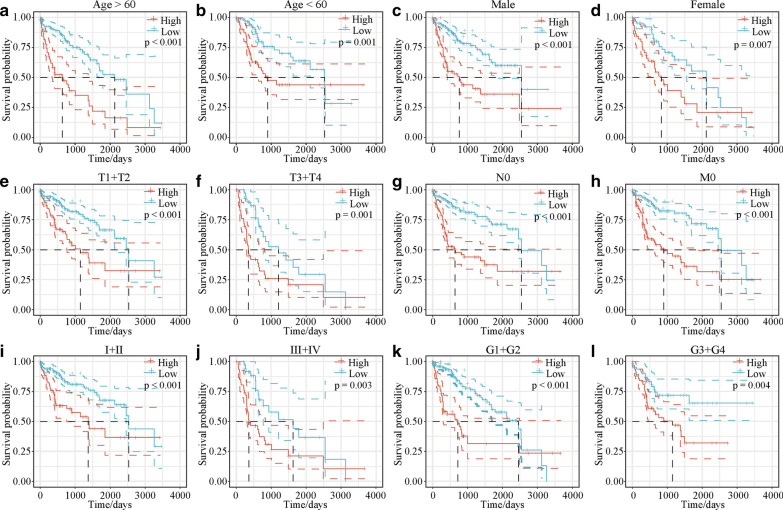
Kaplan–Meier analyses of the five-gene-based model in subgroups with different clinical characteristics. Kaplan–Meier survival plot analyses to assess the efficacy of the five-gene-based model in predicting the survival outcomes of subgroups with different clinical characteristics (age, sex, T1 + T2, T3 + T4, N0, M0, stage I + II, stage III + IV, G1 + G2, and G3 + G4). Kaplan–Meier survival curves for **a** age > 60, **b** age < 60, **c** male, **d** female, **e** T1 + T2, **f** T3 + T4, **g** N0, **h** M0, **i** stage I + II, **j** stage III + IV, **k** G1 + G2, and **l** G3 + G4 for the low-risk and high-risk groups. *T* tumour, *N* node, *M* metastasis, *G* grade

After confirming the efficacy of the five-gene-based model in predicting patient response to immunotherapy, whether the five-gene-based model could be applied to determine the survival outcomes in subgroups with different clinical characteristics was investigated. The results indicated that the five-gene-based model could be used to predict different clinical characteristics (*p* < 0.05; Fig. 5).

### Risk model and prognostic analyses of different gene mutation subtypes

To determine the clinical efficacy of the model in predicting treatment response in HCC patients with somatic mutations, Kaplan–Meier survival analyses were performed. First, 11 genes that are commonly mutated in HCC (tumour protein p53 [*TP53*], phosphatidylinositol-4,5-bisphosphate 3-kinase catalytic subunit alpha [*PIK3CA*], retinoblastoma protein [*RB1*], cyclin-dependent kinase inhibitor 2A [*CDKN2A*], tuberous sclerosis-2 [*TSC2*], β-catenin [*CTNNB1*], AT-rich interactive domain-containing protein 2 [*ARID2*], axin 1 [*AXIN1*], ribosomal protein S6 kinase A3 [*RPS6KA3*], AT-rich interactive domain-containing protein 1A [*ARID1A*], and lysine methyltransferase 2D [*KMT2D*]) were selected. *TP53*, *CTNNB1*, *AXIN1*, and *ARID1A* showed the highest mutation frequencies (28%, 24%, 8% and 7%, respectively) and were therefore used for the Kaplan–Meier analysis (Additional file 3: Figure S2). There was a significant difference in OS between patients in the high-risk and low-risk groups (Additional file 3: Figure S2a with *TP53* mutation; Figure S2b without *TP53* mutation; Figure S2c with *CTNNB1* mutation; and Figure S2d without *CTNNB1* mutation [*p* < 0.05; Additional file 4: Figure S3]). Patients with *AXIN1* or *ARID1A* mutations showed no significant difference in OS between the high-risk and low-risk groups, but a significant difference was observed for patients without *AXIN1* or *ARID1A* mutations between the high-risk and low-risk groups. The results suggest that the five-gene-based model can also be used to predict the survival outcomes of HCC patients with genetic mutations.

### The five-gene-based model can serve as an independent biomarker in clinical applications

**Fig. 6:**
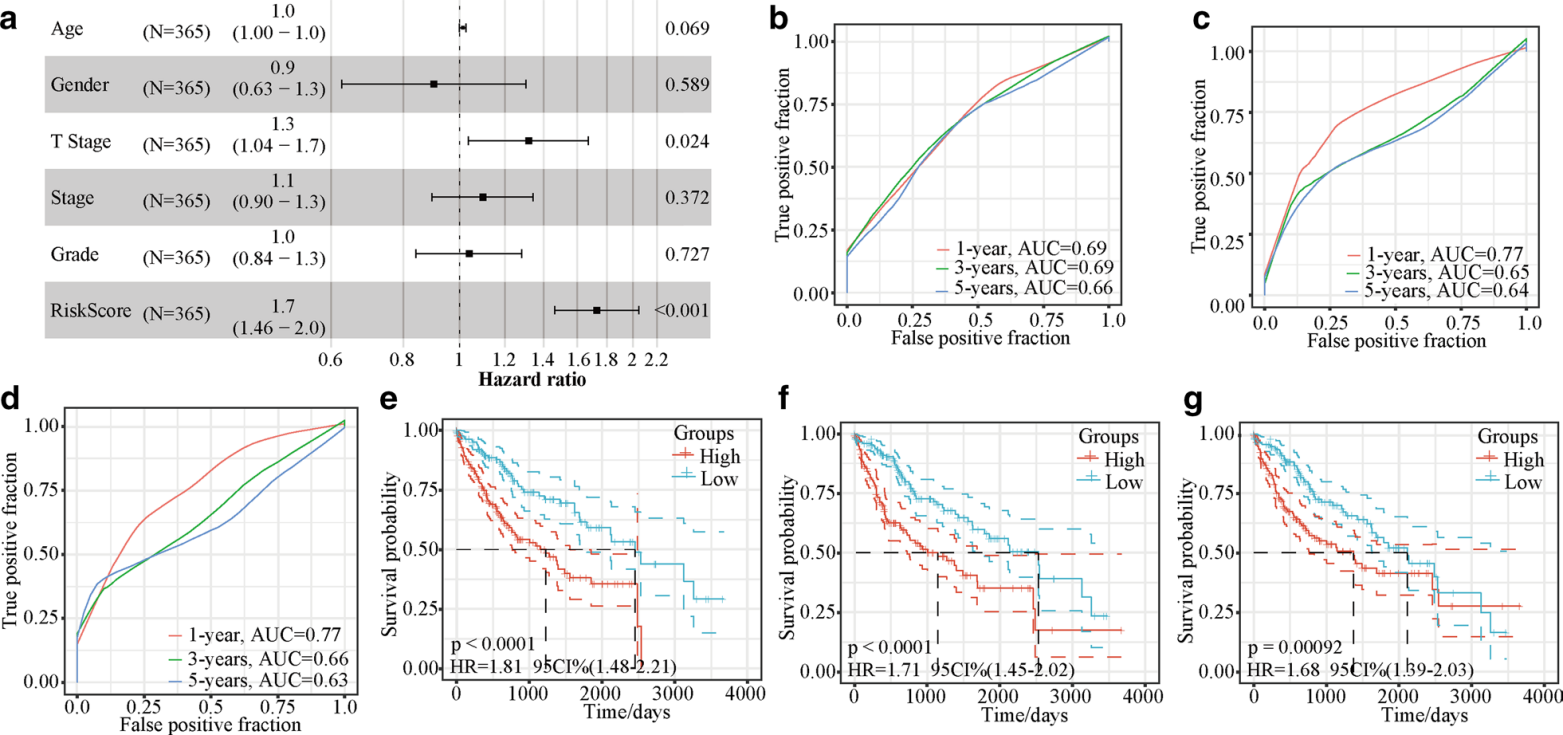
The forest plot and comparison of the five-gene-based model and other models. **a** Forrest plot of the clinicopathological parameters age, T stage, stage, grade, and risk score of 365 HCC patients. Time-dependent ROC analysis and Kaplan–Meier analysis for **b**, **e**. the five-gene signature, **c**, **f**. the HCC prognostic evaluation model, and **d**, **g**. the six-gene model. *ROC* receiver operating characteristic, *HCC* hepatocellular carcinoma, *T* tumour, *AUC* area under the curve, *HR* hazard ratio, *CI* confidence interval

Next, we evaluated whether the five-gene-based model could serve as an independent biomarker with clinical implications. Univariate and multivariate Cox regression analyses of the clinicopathological parameters (including age, sex, T stage, stage, and tumour grade) of 365 patients (Table 2) revealed that the HR of the risk model was approximately 1.7 (*p* < 0.001), and the HR of the T stage model was approximately 1.3 (*p* < 0.001; Fig. 6a). Multivariate Cox regression analysis showed that T stage and the prognostic model were independent risk factors associated with OS.

**Table 2:** Univariate and multivariate analyses of all TCGA datasets

Variables	Univariate analysis				Multivariate analysis			
	HR	95% CI of HR		*P*	HR	95% CI of HR		*P*
		lower	upper			lower	upper	
Age	1.013	0.999	1.027	0.073	1.013	0.999	1.027	0.069
Sex	0.815	0.572	1.161	0.257	0.904	0.626	1.305	0.589
T stage	1.577	1.337	1.860	6.4E-08	1.317	1.037	1.672	0.024
Stage	1.382	1.218	1.569	5.3E-07	1.096	0.896	1.340	0.372
Grade	1.164	0.949	1.427	0.145	1.038	0.841	1.283	0.727
Risk score	1.834	1.564	2.151	8.6E-14	1.730	1.463	2.046	1.5E-10

*HR* hazard ratio, *CI* confidence interval, *TCGA* The Cancer Genome Atlas

### Comparison of the five-gene-based model and other models

Next, the ability of our established model to determine HCC prognosis was compared with those of three other prognostic models: the four-gene signature [23], the HCC prognostic evaluation model [24] and the six-gene signature [25]. We calculated the risk score of the corresponding genes in these three models for the TCGA-LIHC dataset using a method similar to that used for the establishment of our five-gene-based model (described above). The four-gene-based model had lower AUC values for 1-, 3- and 5-year survival rates than our model; the HCC prognostic evaluation model and the six-gene-based model had slightly higher AUC values for the 1-year survival rate than our model but had lower AUC values for 3- and 5-year survival rates (Fig. 6b-d). These results indicated that our model performed better at predicting the long-term survival (3-year and 5-year survival) than the short-term survival (1-year) of HCC patients. Similar to our model, these three models could also predict the OS of the high- and low-risk groups (log-rank *p* < 0.001; Fig. 6e–g).

### Relationship between risk score and KEGG pathways

**Fig. 7:**
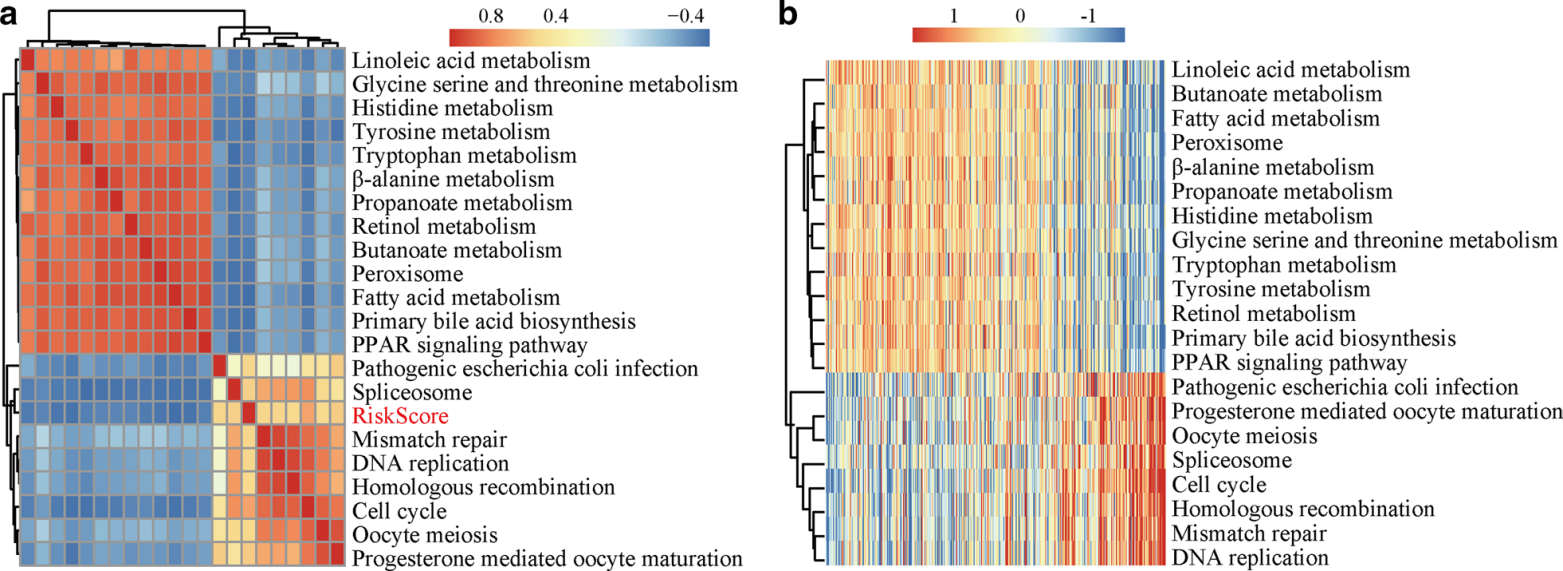
Relationships between the risk score and KEGG pathways. a. Twenty-one KEGG pathways were found to be correlated with the risk score by ssGSEA. b. The ssGSEA score of the KEGG pathway changes as the risk score increases. Samples (rows) are ranked from low to high risk scores. The colour scale indicates upregulation (red) or downregulation (blue) of gene expression. *ssGSEA* single-sample gene set enrichment analysis, *KEGG* Kyoto Encyclopedia of Genes and Genomes

We performed ssGSEA to identify potential KEGG pathways (with a correlation coefficient > 0.45) associated with the risk score. A total of 21 KEGG pathways were identified (Fig. 7a). Analysis of the relationship between gene sets and the risk score revealed that the KEGG pathways positively correlated with the risk score included DNA replication, mismatch repair, cell cycle, homologous recombination, spliceosome, oocyte meiosis, progesterone-mediated oocyte maturation, and pathogenic *Escherichia coli* infection. There were also downregulated pathway terms that negatively correlated with the risk score in HCC, including butanoate metabolism, peroxisome, fatty acid, linoleic acid, tryptophan and tyrosine metabolism (Fig. 7b). The enrichment analysis revealed potential critical pathways implicated in HCC pathogenesis.

## Discussion

Immune subsets were found to be significant effectors of immune defence [26–28]. The use of gene expression signatures to determine the outcomes of treatments has been reported by several groups. We showed that the correlation between gene signatures and the tumour immune microenvironment can be used to predict HCC immunotherapy outcome. Similarly, He et al. [13] established a model based on the expression of immune-related genes to predict treatment outcome, Shen et al. [29] identified a clinical-immune signature to estimate OS in ovarian cancer, and Tekpli et al. [30] discovered a novel independent prognostic factor based on the tumour immune microenvironment in breast cancer. Recently, integrative analysis of multi-omics data revealed epidermal growth factor receptor (EGFR) as a critical node in the gene regulatory network that is related to immune phenotype, and the inclusion of therapeutic EGFR inhibition enhanced head and neck squamous cell carcinoma patient response to ICIs [31]. This finding suggests that critical genes associated with the tumour immune microenvironment may serve as prognostic signatures and be useful for clinical immunotherapy.

HCC is a highly heterogeneous tumour at the molecular level and is pathological [32]. The most frequent mutations were identified in telomerase reverse transcriptase (*TERT*), *ARID1A*, *TP53*, and *CTNNB1* [33–37]. These gene mutations were also associated with cell differentiation, proliferation, and clinical features [32, 38]. In our study, the five identified genes had a low frequency of mutations, and they were conducive to constructing a stable prognostic model. In addition, the five-gene-based model established in the present study could predict HCC treatment outcomes for patients with or without tumour-specific mutations in the *TP53*, *CTNNB1*, *AXIN1* or *ARID1A* genes. Thus, the five-gene-based prognostic model was a useful classification tool for patients with various genetic mutation backgrounds.

TMB is an emerging biomarker of sensitivity to immunotherapy [39, 40]. Cai et al. [41] showed that high TMB in liver cancer patients with radical resection was significantly correlated with poor prognosis. Stenzinger et al. [42] reported that high TMB correlated with increased patient response rates and survival benefits from immune checkpoint inhibitors. Tumour-specific NEO that form as a consequence of mutations are thought to be another important biomarker for the therapeutic efficacy of cancer immunotherapies [43, 44]. In our study, ROC curve analysis showed that the combined consideration of NEO burden, TMB and risk score output a higher AUC value than NEO burden, TMB, or risk score alone. Combining the five-gene-based model with NEO burden and TMB could enhance the assessment of immunotherapy efficacy and identify patients who will respond to immunotherapy. In the present study, we identified five genes (*LDHA*, *PPAT*, *BFSP1*, *NR0B1*, and *PFKFB4*) associated with the tumour immune microenvironment in HCC patient cohorts that may be applied as potential biomarkers of HCC immunotherapy outcome. The correlation between the risk score and HCC prognosis was determined by analysing the mRNA expression and protein expression of the identified genes with an HCC TMA by qPCR and IHC, respectively. We showed for the first time that *BFSP1* is correlated with HCC outcomes. PPAT catalyses the first committed step of de novo purine nucleotide biosynthesis [45, 46], suggesting that targeting PPAT might be a promising cancer strategy [47]. PPAT was also identified as a prognostic biomarker in HCC [48]. Many studies have reported that upregulated LDHA promotes tumour metastasis and is correlated with poor prognosis in several cancers, including lung adenocarcinoma [49], breast cancer [50], HCC [51], gallbladder carcinoma [52], and renal cell carcinoma [53]. Since high LDHA expression can reduce the oxygen dependency of tumour cells by promoting efficient anaerobic/glycolytic metabolism, targeting LDHA is a potential anti-tumour strategy. Epigenetic modification of *NR0B1* leads to its ectopic activation in Ewing's sarcoma and lung cancer, enabling it to promote cancer cell proliferation [54–57]. The overexpression of PFKFB4 was found to be associated with a poor prognosis in gastric cancer [58], bladder cancer [59], colon cancer [60], acute monocytic leukaemia [61], glioblastoma [62], thyroid cancer [63], and breast cancers [64–66]. A better understanding of the molecular mechanisms underlying the ability of the five-gene signature to predict HCC pathogenesis as well as prospective studies to validate its utility in clinical applications are needed for the further development of new therapeutic and prognostication strategies (Additional file 5: Table S2).

In this work, we established a prognostic signature for HCC OS prediction that also effectively predicts the efficacy of immunotherapy through combined analysis of gene expression datasets from GEO and TCGA. The model was based on gene mRNA expression but not gene mutations or epigenetic modifications of these five genes. Therefore, it has good clinical feasibility, as it does not require whole-genome sequencing for all patients. Moreover, the methods used in this study might also be suitable for other types of malignancies. In further studies, we plan to detect the expression of these five genes in circulating tumour cells. However, there are several limitations in our study. First, the prognostic role of the five genes at the protein level warrants further research. Second, the model was established with tumour tissues, so it can only predict the prognosis of HCC patients after surgery and cannot detect and diagnose tumours at the early stage. Third, further functional experiments are needed, and the underlying mechanism of the five genes needs to be clarified (Additional file 6).

## Conclusions

In this study, we established a novel five-gene-based prognostic model based on the tumour immune microenvironment. Importantly, our model could effectively predict the efficacy of immunotherapy and might serve as a potential independent biomarker in clinical applications.

## Supplementary Information

**Additional file 1: Table S1.** Primary antibody information.

**Additional file 2: Figure S1.** Output profiles from the analyses of the TCGA validation set and entire TCGA dataset based on the five-gene-based HCC prognostic model. Risk score (top left), patient status (top right), mRNA expression heatmap (bottom left), and time-dependent ROC and Kaplan-Meier curves (bottom right) of the five-gene-based model for S1a. the TCGA-LIHC validation set and S1b. the entire TCGA dataset. Abbreviations: TCGA, The Cancer Genome Atlas; HCC, hepatocellular carcinoma; ROC, receiver operating characteristic; PPAT, phosphoribosyl pyrophosphate amidotransferase; BFSP1, beaded filament structural protein 1; LDHA, lactate dehydrogenase A; NR0B1, nuclear receptor subfamily 0 group B member 1; PFKFB4, 6-phosphofructo- 2-kinase/fructose-2,6-bisphosphatase 4.

**Additional file 3: Figure S2.** Genes showing the highest mutation frequency in the TCGA-LIHC dataset. Abbreviations: TP53, tumour protein p53; PIK3CA, phosphatidylinositol-4,5-bisphosphate 3-kinase catalytic subunit alpha; RB1, retinoblastoma protein; CDKN2A, cyclin-dependent kinase inhibitor 2A; TSC2, tuberous sclerosis-2; CTNNB1, β-catenin; ARID2, AT-rich interactive domain-containing protein 2; AXIN1, axin 1; RPS6KA3, ribosomal protein S6 kinase A3; ARID1A, AT-rich interactive domain-containing protein 1A; KMT2D, lysine methyltransferase 2D.

**Additional file 4: Figure S3.** Kaplan-Meier survival analysis to assess the ability of the five-gene-based model to predict HCC therapy outcome for patient groups a. with TP53 mutation, b. without TP53 mutation, c. with CTNNB1 mutation, and d. without CTNNB1 mutation. Abbreviations: HCC, hepatocellular carcinoma; TP53, tumour protein p53; CTNNB1, β-catenin; AXIN1, axin 1; ARID1A, AT-rich interactive domain-containing protein 1A.

**Additional file 5: Table S2.** The sample information of TCGA training set and validation set.

**Additional file 6. **Additional material.

## Data Availability

All data in our study are available upon request.

## Abbreviations

LASSO: Least absolute shrinkage and selection operator
OS: Overall survival
TCGA-LIHC: The Cancer Genome Atlas liver hepatocellular carcinoma
HCC: Hepatocellular carcinoma
PFKFB4: 6-Phosphofructo-2-kinase/fructose-2,6-bisphosphatase 4
PPAT: Phosphoribosyl pyrophosphate amidotransferase
BFSP1: Beaded filament structural protein 1
LDHA: Lactate dehydrogenase A
NR0B1: Nuclear receptor subfamily 0 group B member 1
IHC: Immunohistochemistry
ROC: Receiver operating characteristic
NEO: Neoantigens
TMB: Tumour mutational burden
CR: Complete response
PR: Partial response
SD: Stable disease
PD: Progressive disease
IC: Immune cell
TC: Tumour cell
T: Tumour
N: Node
M: Metastasis
G: Grade
ssGSEA: Single-sample gene set enrichment analysis
KEGG: Kyoto Encyclopedia of Genes and Genomes
