# Supplementary material for: Model based on five tumour immune microenvironment-related genes for predicting hepatocellular carcinoma immunotherapy outcomes
Source: J Transl Med. 2021 Jan 6;19:26. doi: 10.1186/s12967-020-02691-4 (PMC7788940; doi:10.1186/s12967-020-02691-4)
Supplement: Supplementary file 1 — Additional file 1: Table S1. Primary antibody information. [file 12967_2020_2691_MOESM1_ESM.docx]

**Table S1.** Primary antibody information.

| **Primary antibody** | **Supplier** | **Product code** | **Dilution factor** |
| --- | --- | --- | --- |
| Anti-PPAT | Atlas  Antibodies AB | HPA036091 | 1:25 |
| Anti-BFSP1 | Atlas  Antibodies AB | HPA042038 | 1:500 |
| Anti-NR0B1 | Abcam | ab196649 | 1:100 |
| Anti-PFKFB4 | Abcam | ab137785 | 1:3,000 |
| Anti-LDHA (C4B5) | CST | 3582S | 1:3,500 |
