# Supplementary figures and images for: Model based on five tumour immune microenvironment-related genes for predicting hepatocellular carcinoma immunotherapy outcomes
Source: J Transl Med. 2021 Jan 6;19:26. doi: 10.1186/s12967-020-02691-4 (PMC7788940; doi:10.1186/s12967-020-02691-4)

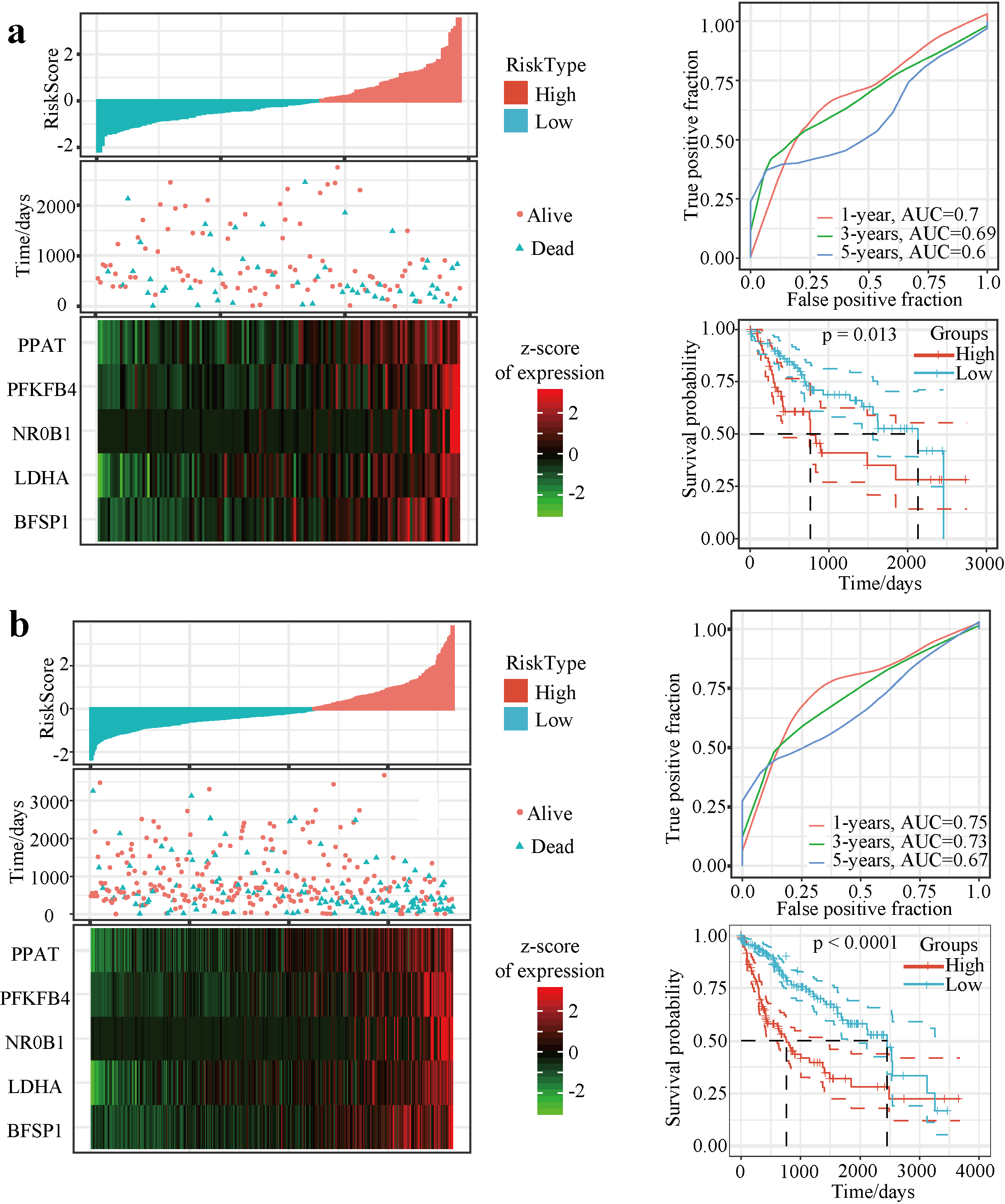

Supplement: Supplementary file 2 — Additional file 2: Figure S1. Output profiles from the analyses of the TCGA validation set and entire TCGA dataset based on the five-gene-based HCC prognostic model. Risk score (top left), patient status (top right), mRNA expression heatmap (bottom left), and time-dependent ROC and Kaplan-Meier curves (bottom right) of the five-gene-based model for S1a. the TCGA-LIHC validation set and S1b. the entire TCGA dataset. Abbreviations: TCGA, The Cancer Genome Atlas; HCC, hepatocellular carcinoma; ROC, receiver operating characteristic; PPAT, phosphoribosyl pyrophosphate amidotransferase; BFSP1, beaded filament structural protein 1; LDHA, lactate dehydrogenase A; NR0B1, nuclear receptor subfamily 0 group B member 1; PFKFB4, 6-phosphofructo- 2-kinase/fructose-2,6-bisphosphatase 4. [file 12967_2020_2691_MOESM2_ESM.tif]

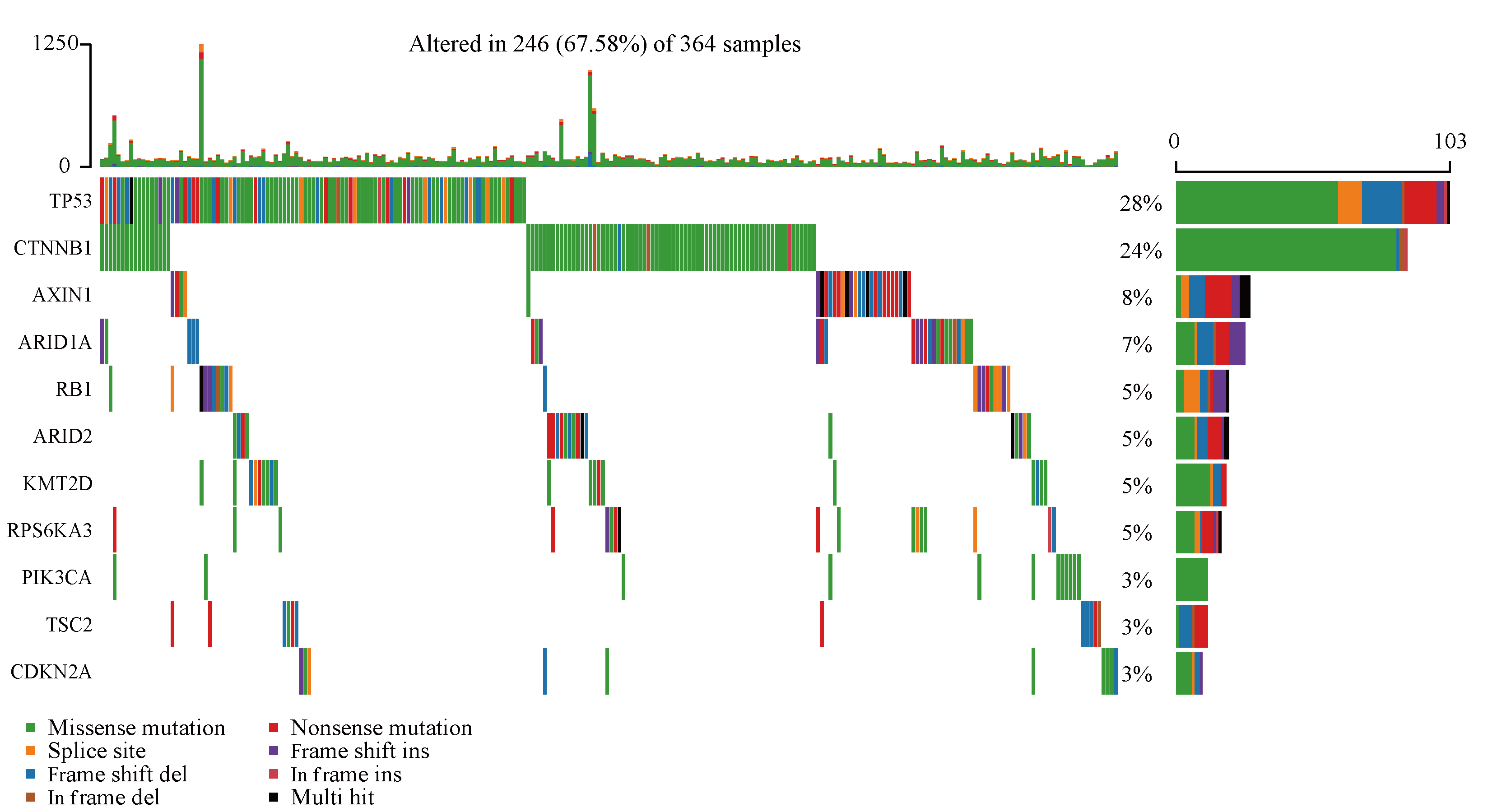

Supplement: Supplementary file 3 — Additional file 3: Figure S2. Genes showing the highest mutation frequency in the TCGA-LIHC dataset. Abbreviations: TP53, tumour protein p53; PIK3CA, phosphatidylinositol-4,5-bisphosphate 3-kinase catalytic subunit alpha; RB1, retinoblastoma protein; CDKN2A, cyclin-dependent kinase inhibitor 2A; TSC2, tuberous sclerosis-2; CTNNB1, β-catenin; ARID2, AT-rich interactive domain-containing protein 2; AXIN1, axin 1; RPS6KA3, ribosomal protein S6 kinase A3; ARID1A, AT-rich interactive domain-containing protein 1A; KMT2D, lysine methyltransferase 2D. [file 12967_2020_2691_MOESM3_ESM.tif]

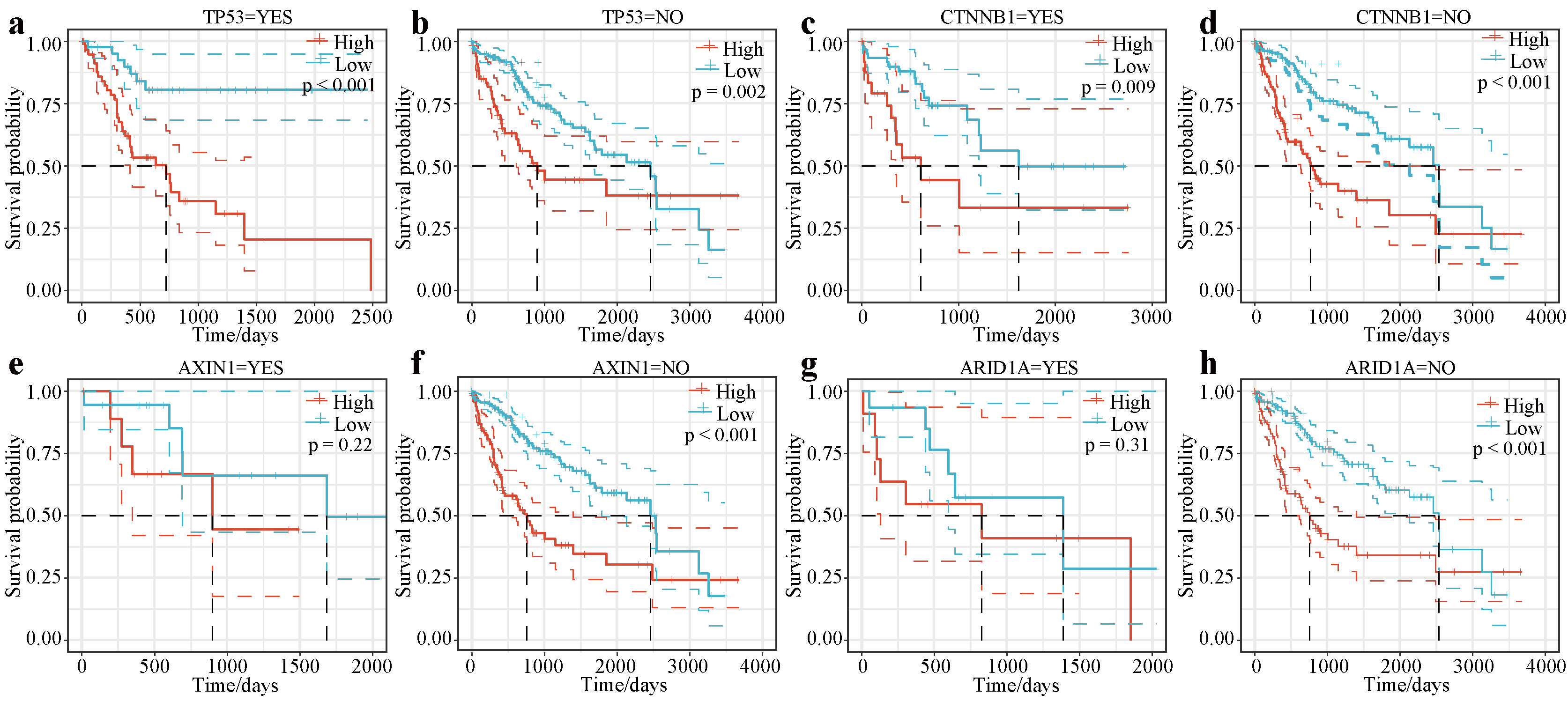

Supplement: Supplementary file 4 — Additional file 4: Figure S3. Kaplan-Meier survival analysis to assess the ability of the five-gene-based model to predict HCC therapy outcome for patient groups a. with TP53 mutation, b. without TP53 mutation, c. with CTNNB1 mutation, and d. without CTNNB1 mutation. Abbreviations: HCC, hepatocellular carcinoma; TP53, tumour protein p53; CTNNB1, β-catenin; AXIN1, axin 1; ARID1A, AT-rich interactive domain-containing protein 1A. [file 12967_2020_2691_MOESM4_ESM.tif]
