# Supplementary material for: Model based on five tumour immune microenvironment-related genes for predicting hepatocellular carcinoma immunotherapy outcomes
Source: J Transl Med. 2021 Jan 6;19:26. doi: 10.1186/s12967-020-02691-4 (PMC7788940; doi:10.1186/s12967-020-02691-4)
Supplement: Supplementary file 5 — Additional file 5: Table S2. The sample information of TCGA training set and validation set. [file 12967_2020_2691_MOESM5_ESM.docx]

**Table S2. The sample information of TCGA training set and validation set**

| **Clinical Features** | **TCGA-LIHC train** | **TCGA-LIHC test** | **P** |
| --- | --- | --- | --- |
| **OS** |  |  |  |
| 0 | 144 | 91 | 0.577 |
| 1 | 75 | 55 |  |
| **T Stage** |  |  |  |
| T1 | 104 | 76 | 0.9402 |
| T2 | 56 | 35 |  |
| T3 | 49 | 29 |  |
| T4 | 8 | 5 |  |
| TX | 2 | 1 |  |
| **N Stage** |  |  |  |
| N0 | 151 | 97 | 0.6889 |
| N1 | 3 | 1 |  |
| NX | 65 | 48 |  |
| **M Stage** |  |  |  |
| M0 | 167 | 96 | 0.07748 |
| M1 | 2 | 1 |  |
| Mx | 50 | 49 |  |
| **Stage** |  |  |  |
| Ⅰ | 97 | 73 | 0.4286 |
| Ⅱ | 52 | 32 |  |
| III | 56 | 27 |  |
| Ⅳ | 2 | 2 |  |
| X | 12 | 12 |  |
| **Grade** |  |  |  |
| G1 | 30 | 25 | 0.901 |
| G2 | 105 | 70 |  |
| G3 | 74 | 44 |  |
| G4 | 7 | 5 |  |
| GX | 3 | 2 |  |
| **Gender** |  |  |  |
| Male | 146 | 100 | 0.802 |
| Female | 73 | 46 |  |
| **Age** |  | 0 |  |
| ≤ 60 | 108 | 65 | 0.4285 |
| ＞60 | 111 | 81 |  |
